# The evolution of gestation length in eutherian mammals

**DOI:** 10.1098/rspb.2024.1412

**Published:** 2024-10-30

**Authors:** Thodoris Danis, Antonis Rokas

**Affiliations:** ^1^ Department of Biological Sciences, Vanderbilt University, Nashville, TN 37235, USA; ^2^ Evolutionary Studies Initiative, Vanderbilt University, Nashville, TN 37235, USA

**Keywords:** gestation length, body mass, lifespan, life-history trait, adaptive shift, allometry, macroevolution

## Abstract

Eutherian mammals exhibit considerable variation in their gestation lengths, which has traditionally been linked to variation in other traits, including body mass and lifespan. To understand how gestation length variation, including its association with body mass and lifespan variation, changed over mammalian evolution, we conducted phylogeny-informed analyses of 845 representative extant species. We found that gestation length substantially differed in both whether and how strongly it was associated with body mass and lifespan across mammals. For example, gestation length was not associated with lifespan or body mass in Chiroptera and Cetacea but was strongly associated only with body mass in Carnivora. We also identified 52 evolutionary shifts in gestation length variation across the mammal phylogeny and 14 shifts when we jointly considered variation of all three traits; six shifts were shared. Notably, two of these shifts, both positive, occurred at the roots of Cetacea and Pinnipedia, respectively, coinciding with the transition of these clades to the marine environment, whereas a negative shift occurred at the root of Chiroptera, coinciding with the evolution of flight in this clade. These results suggest that the relationship between gestation length and the two other traits has varied substantially across mammalian phylogeny.

## 1. Introduction

Life-history traits encompass a group of coexisting and frequently coevolving characteristics that describe organisms’ patterns of survival and reproduction. These patterns significantly contribute to their overall fitness [1]. Life-history traits include lifespan, the number and the sex ratio of offspring, gestation length and age at the weaning stage. Within the realm of mammalian reproductive biology, gestation length holds particular significance, contributing to lifetime reproductive success and offspring survival [2,3]. Eutherian mammals exhibit considerable variation in their gestation length [2,4–8], a trait that has traditionally been linked to body mass and multiple life-history traits, such as lifespan [2,4,7,9–14]. While lifespan and gestation are correlated with each other and with body mass, studying them together allows us to explore broader patterns of life-history evolution in mammals. By considering multiple traits simultaneously, we aim to uncover potential interrelationships and trade-offs that shape reproductive strategies.

Studies involving relatively small numbers of mammals have consistently found linear correlations between body mass and diverse life-history traits, including gestation length, suggesting trait–trait coevolution and that some of the genetic loci that contribute to the variation of these traits may be shared [4,7,12,15]. The advent of powerful analytical strategies that incorporate phylogenetic information [16–23] and the assembly of increasingly comprehensive databases of life-history traits for an increasing number of eutherian mammal species have expanded our understanding of how ecological shifts have affected the evolution of these traits. Previous research has focused primarily on relating gestation length, lifespan, metabolic rate and neonatal status to body mass and brain size [4,6,14,24–26]. More recent studies have investigated embryonic development, reproduction rates and diet, revealing links to various co-factors in mammals [27–29]. Some have even explored the association between cancer risk in mammals and reproductive life-history traits, such as litter size and gestation length, or reproductive developmental features, such as placental development, to cancer risk in mammals [27].

In this study, we extend and build upon the findings of previous comparative investigations [4,7,10,12,25,30–32] by analysing a much larger dataset of gestation length, body mass and lifespan data from 845 diverse eutherian mammals to comprehensively examine: (i) lineage-specific patterns of gestation length evolution and the extent to which gestation length is correlated—after accounting for phylogeny—with the evolution of body mass and lifespan; (ii) evolutionary or adaptive shifts in the evolution of gestation length; and (iii) shifts in the joint evolution of gestation length, body mass and lifespan.

## 2. Results and discussion

### (a) Differential associations of gestation length with body mass and lifespan across mammals

To understand the evolution of gestation length across mammals and how it has been influenced by other major traits, we collected data on gestation length, body mass and lifespan for 845 eutherian mammals from the PanTHERIA [33], AnAge [34], EltonTraits [35] and MOM-Mammals [36] databases. All three traits exhibit extensive variation; the coefficient of variation for gestation length is 19.98%, for lifespan is 33.09% and for body mass is 44.24% (electronic supplementary material, table S1).

**Figure 1.**
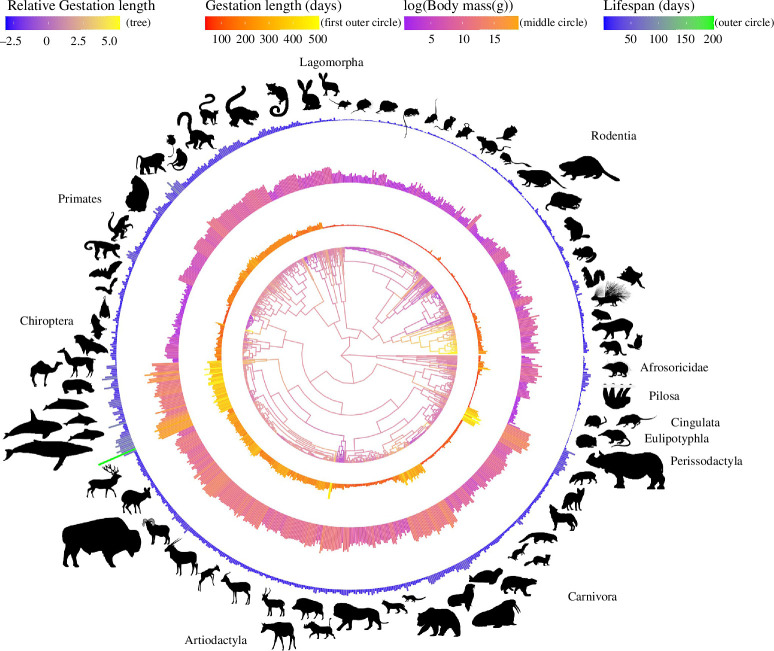
Variation in gestation length, body mass and lifespan across 845 eutherian mammals. The colours of the branches of the phylogenetic tree illustrate relative gestation length, which was inferred by employing ancestral reconstruction on the residuals from the phylogenetic generalized least squares (PGLS) regression of log(gestation length) ~ log(body mass) + log(lifespan) + log(body mass × lifespan). The three outer circles depict gestation length (absolute values, in days), body mass (log-transformed, in grams) and lifespan (absolute values, in days), arranged from the innermost to the outermost circle. Decisions on showing absolute versus log-transformed values were made for better visualization. The data used to draw this figure are available in electronic supplementary material, data S1. Silhouette illustrations are from phylopic.org. Phylogeny is from Upham *et al*. [37].

To isolate the influence of gestation length independent of body size and lifespan, we first reconstructed the relative gestation length using the residuals from the model of log(gestation length) ~ log(body mass) + log(lifespan) + log(body mass × lifespan) (figure 1; tree) and then mapped the quantitative variation of all three traits onto a time-calibrated mammal phylogeny [37]. We found major differences in the patterns of variation of all three traits (figure 1). The mapping of gestation length onto a time-calibrated phylogeny revealed that both the absolute and the relative gestation lengths of different groups of mammals changed multiple times independently. These changes in gestation length appear closely related to changes in absolute body mass values (figure 1). For example, in the taxonomic orders of Rodentia and Perissodactyla, the gestation length and lifespan were generally consistent, with the gestation length showing slightly less variation than lifespan within Rodentia (electronic supplementary material, table S1). In contrast, within Carnivora, only Pinnipedia exhibits extended gestation lengths and lifespans, while the remaining taxa within Carnivora demonstrate moderate variability in these traits (figure 1; electronic supplementary material, table S1). Longer gestation lengths, larger body masses and extended lifespans are also observed in Cetacea (figure 1). These findings align with observations from previous studies [4,7,8,12,25].

To better understand the relationship between gestation length and the other two traits, we performed phylogenetic regression of gestation length with body mass and lifespan as covariates using Pagel’s model [23]. Examination of variance inflation factors (VIFs) revealed that our parameter estimates were generally unaffected by multicollinearity, except for Cetacea and Eulipotyphla, which exhibited elevated VIF values of 3.08 and 3.19, respectively, but remained below the commonly used threshold of 5.00 [38,39] (table 1).

**Table 1.** Results of Pagel’s model fitting of log(gestation length) ~ log(body mass) + log(lifespan) + log(body mass × lifespan). Bold cells indicate significant *p* values (<0.05). Only clades with more than 20 species are reported. VIF, variance inflation factor.

clades	#species	Pagel’s *λ*	intercept	slope body mass	slope lifespan	slope (body mass × lifespan)	*p*‐value (body mass)	*p*‐value (lifespan)	*p*‐value (body mass × lifespan)	VIF	*R* ^2^
**Rodentia**	220	0.83	3.70	0.11	0.04	0.030	**0.01**	0.18	**0.03**	1.71	0.85
**Artiodactyla (excluding Cetacea)**	156	1.00	5.33	0.04	0.08	0.050	**0.01**	0.35	0.11	1.64	0.73
**Primates**	128	1.00	5.00	0.09	0	0	**0.01**	0.96	0.96	2.04	0.75
**Carnivora**	121	0.89	4.29	0.13	0.09	0.070	**0.01**	**0.03**	**0.02**	1.65	0.73
**Primates** **(Old World monkeys (OWM))**	53	0.66	5.27	0.03	0.04	0.120	0.17	0.4	**0.02**	1.45	0.68
**Cetacea**	44	0.96	5.88	0.08	0.03	−0.008	**0.04**	**0.05**	0.50	3.08	0.47
**Primates** **(New World monkeys (NWM))**	40	0.29	5.06	0.12	−0.02	0.060	**0.01**	0.63	0.14	1.45	0.71
**Chiroptera** **(Yangochiroptera)**	33	1.00	4.59	0.08	−0.02	−0.004	0.29	0.87	0.97	1.00	0.60
**Eulipotyphla**	35	0	3.36	0.14	0.10	−0.060	**0.01**	0.10	**0.06**	3.19	0.71
**Prosimians**	35	1.00	4.99	0.10	−0.06	−0.090	**0.01**	0.63	0.33	0.57	0.75
**Pinnipedia**	26	1.00	5.32	0.06	−0.47	−0.010	0.24	**0.02**	0.94	1.05	0.14
**Chiroptera** **(Yinpterochiroptera)**	28	0	4.89	0.07	−0.12	0.040	0.16	0.16	0.37	1.61	0.54

We observed significant variations in both whether gestation length was associated with body mass and lifespan across different mammalian lineages, and in the strength of these associations (table 1). Specifically, there were no significant associations between gestation length and the two covariates, body mass and lifespan, or their interaction in Chiroptera (table 1). In contrast, both NWM and OWM (Primates) displayed significant associations between gestation length and body mass, as well as an interaction effect between the two covariates (table 1). Finally, there was a significant association between gestation length and body mass in Rodentia, Cetacea, Carnivora and Artiodactyla (excluding Cetacea; table 1).

**Figure 2.**
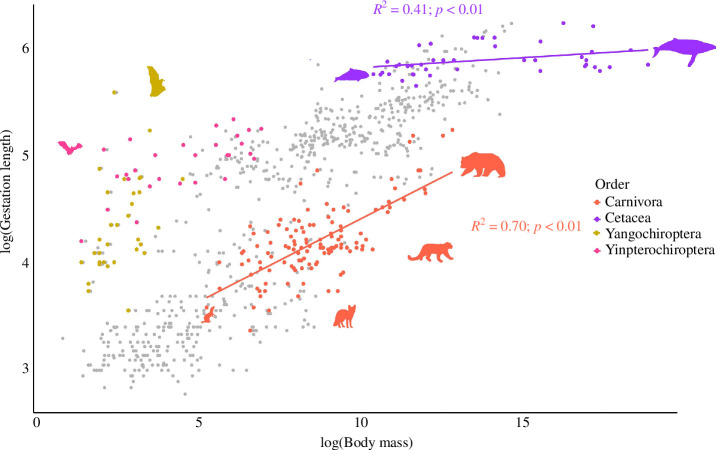
Variation in gestation length differs substantially in both whether and how strongly it is associated with body mass across mammals. The scatterplot shows the relationship between gestation length and body mass across eutherian mammals, with each dot corresponding to one of the mammalian species used in our study. Phylogenetic regression analysis using Pagel’s model [23] was performed on four taxa from three mammalian orders: Carnivora, Cetacea and Chiroptera (Yangochiroptera and Yinpterochiroptera). Each coloured data point represents a species within these taxa; data points in grey correspond to species from the rest of eutherian mammals. The *R*^2^ values show the proportion of variance explained by the model log(gestation length) ~ log(body mass) in Carnivora and Cetacea, the two clades with significant *p*-values; the two traits are not significantly associated with Yangochiroptera and Yinpterochiroptera. Silhouette illustrations are from phylopic.org. The results of all eutherian taxa tested under the log(gestation length) ~ log(body mass) model are shown in electronic supplementary material, figure S1 and table S2.

We also performed phylogenetic regression of gestation length with body mass (figure 2; electronic supplementary material, table S2). In the log(gestation length) ~ log(body mass) model (electronic supplementary material, table S2), body mass was found to be a significant predictor of gestation length in several clades, with particularly strong explanatory power in Rodentia (*R*^2^ = 0.86), Artiodactyla (*R*^2^ = 0.72), Carnivora (*R*^2^ = 0.70) and Primates (NWM; *R*^2^ = 0.70). Other clades such as Lagomorpha (*R*^2^ = 0.64), Eulipotyphla (*R*^2^ = 0.61) and Primates (OWM; *R*^2^ = 0.64) also showed significant relationships with body mass, explaining a substantial portion of the variance in gestation length. However, we also found several clades where the relationship between gestation length and body mass was not significant, such as Chiroptera, Pinnipeds, Cingulata, Pilosa, Afrosoricida and Perissodactyla.

The varying significant associations observed across different clades in our study challenge the notion that gestation length scales uniformly with body mass and other life-history traits across mammals [7,12,14,40,41] (table 1). These findings are consistent with previous studies using smaller numbers of taxa [1,7,10,12,14,28] and different combinations of life-history traits [4,7,12,15,25]. Rather, our results raise the hypothesis that different taxa may have experienced differing and varying levels of selection pressure on gestation length and/or on its linkage to other life-history traits [4]. Examination of the varying association of gestation length with other life-history traits and its potential connection to ecological changes or historical events that differentially affected different mammalian taxa represents a very interesting topic for future studies.

### (b) Multiple evolutionary shifts in gestation length in mammalian evolution

**Figure 3.**
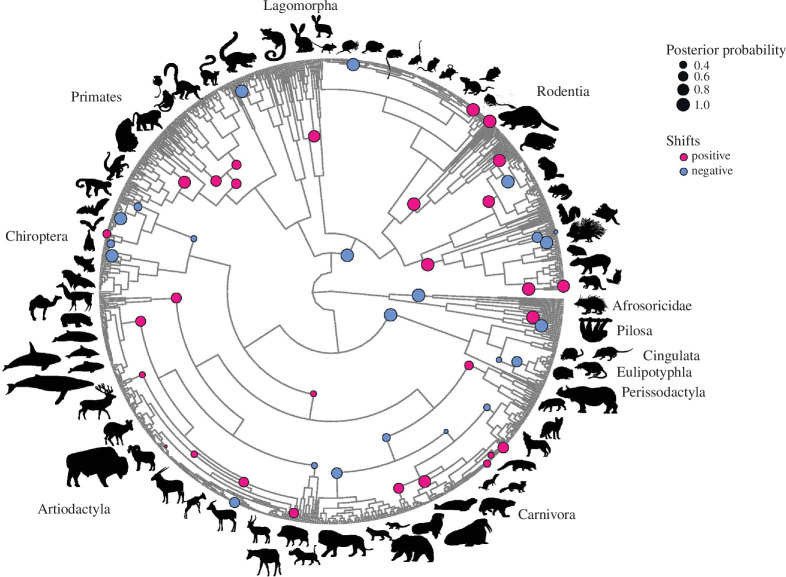
Changes in the phenotypic optimum of gestation length (evolutionary shifts) have been frequent in mammalian evolution. Circles denote the placements and magnitudes of the 52 evolutionary shifts in gestation length inferred by the Bayou Bayesian method [43]. The colour of each circle corresponds to the direction of the evolutionary shift (positive shifts, *n* = 29, dark pink; negative shifts, *n* = 23, blue) and the size of each circle to the posterior probability of the shift; only shifts with a posterior probability greater than 0.25 are included. Silhouette illustrations are from phylopic.org. Phylogeny is from Upham *et al*. [37].

Changes in the environment or in selective pressures can drive modifications of phenotypic traits, leading to adaptive or evolutionary shifts [42]; these shifts are best interpreted as changes in trait optimal values [43]. To deepen our understanding of the evolution of gestation length and identify its shifts across the eutherian mammal phylogeny, we first performed a univariate Bayesian analysis of gestation length. We found 52 shifts with posterior probabilities greater than 0.25 in the evolution of gestation length across eutherian mammals. Of these 52 shifts, 29 were positive and increased gestation length, and the remaining 23 were negative and decreased gestation length (figure 3).

Most of the shifts occur within mammalian orders, except for a negative shift at the root of Rodentia and Lagomorpha. The number and direction of these shifts differ across orders. For example, Primates exhibit four positive shifts early in their evolutionary history, Rodentia contain eight positive and five negative evolutionary shifts and Artiodactyla (excluding Cetacea) exhibit one negative and six positive shifts. In Chiroptera (bats), four negative shifts and one positive shift were observed in Yangochiroptera and one negative shift in Yinpterochiroptera. Bats are the only mammals capable of powered flight [44,45], which imposes unique physiological and metabolic demands [46–49]. We hypothesize that the metabolic cost of flight may have contributed to the evolution of shorter gestation lengths in bats.

Interestingly, there were positive shifts toward longer gestation lengths at the base of Pinnipedia and Cetacea, two taxa that independently transitioned to the marine environment. The evolution of cetaceans from terrestrial artiodactyl ancestors to fully aquatic organisms has been one of the most dramatic ecological transitions [50–53]. This shift resulted in profound morphological, physiological and reproductive adaptations. Changes in gestation length towards longer periods may be a response to the complex requirements of marine life. Offspring need to be mature at birth to navigate an aquatic environment [51,54]. The evolution of longer gestation periods would ensure that the mother has sufficient energy reserves to support both herself and her fetus. Significant changes in physiology and reproductive strategies also occurred during the evolutionary transition of pinnipeds, such as sea lions and seals, to their semi-aquatic lifestyle. In this semi-aquatic environment, newborns must be capable of both terrestrial and aquatic mobility soon after birth, which necessitates a high level of developmental maturity at birth [55–57]. Therefore, the evolution of longer gestation periods would ensure that offspring are born at an advanced developmental stage, enhancing their chances of survival. Additionally, an extended maternal investment window before birth could be beneficial by providing the mother with more time to seek more favourable conditions, such as food and safe birthing locations [53,57].

**Figure 4.**
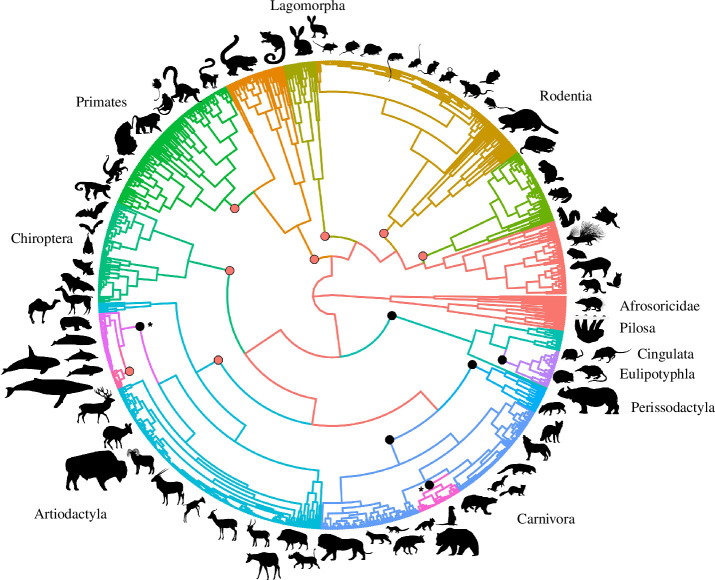
Joint consideration of gestation length, body mass and lifespan reveals multiple evolutionary shifts during mammalian evolution. Circles denote the placements of the 14 shifts when jointly considering the evolution of gestation length, lifespan and body mass across mammals using the PhylogeneticEM method [42,58]. The six evolutionary shifts common to this analysis and the analysis reported in figure 3 are coloured black. The two asterisks correspond to the evolutionary shifts at the bases of the Cetacea and Pinnipedia clades. Branches are coloured for easier visualization of the lineages that experienced evolutionary shifts. Silhouette illustrations are from phylopic.org. Phylogeny is from Upham *et al*. [37].

Given that the evolution of gestation length is correlated with the evolution of body mass and/or lifespan in several taxa (table 1; figure 2; electronic supplementary material, figure S1 and table S2), we also conducted a multivariate analysis that jointly considered the evolution of these three variables across eutherian mammals. This analysis revealed 14 shifts (figure 4; electronic supplementary material, table S3), most of which occurred at or near the roots of different orders and sustained their positive trend. Three of the shifts occurred at the roots of the orders Artiodactyla, Chiroptera and Primates. For Chiroptera, the shift largely reflected a decrease in body mass, whereas gestation length and lifespan remained relatively unchanged. Furthermore, 6 of the 14 shifts overlap with shifts also found in our univariate analysis of gestation length (figure 3). Interestingly, two of these shared shifts occurred at the roots of the Cetacea and Pinnipedia clades and, in both cases, involved increases in all three traits, indicating a sustained evolutionary trend towards larger body masses, longer lifespans and extended gestation periods. For Pinnipedia, body mass increased roughly seven times as much as lifespan and approximately 2.7 times as much as gestation length (estimates based on the unit changes observed in the traits’ theta values). For Cetacea, body mass increased roughly four times more than lifespan and gestation length. There was an additional shift in our multivariate analysis within the Mysticeti, where body mass increased more than four times as much as lifespan, but gestation length remained unchanged.

We did not observe any evolutionary shifts in our multivariate analysis where changes in gestation length were greater in magnitude than changes in the other two traits. In general, the greatest shifts in trait values occurred for body mass, followed by gestation length and lifespan. These results suggest that gestation length coevolved with body mass and lifespan in some eutherian mammal taxa but appears to have evolved independently of the other two covariates in others [4,7,25].

The univariate analysis of gestation length using the Bayou Bayesian method [43] (figure 3) and the multivariate analysis of gestation length, body mass and lifespan using the PhylogeneticEM method [42,58] (figure 4) yielded different numbers of evolutionary shifts, with some overlap. This is not surprising given that the univariate analysis aims to identify changes in gestation length optima, whereas the multivariate approach aims to identify concurrent changes in the optima of all three analysed traits. This difference in experimental design results in fewer shifts observed with the multivariate method because inference of evolutionary shifts is based on the combined changes across all three traits on a given branch rather than on each individual trait. On the one hand, inference of shifts based on concurrent changes in all three traits by the PhylogeneticEM analysis may mask any asynchronous shifts in individual traits, contributing to the observed differences in the numbers of shifts identified by the two methods. On the other hand, the PhylogeneticEM method [20,23] uniquely captures coevolutionary dynamics between traits, revealing novel shifts not solely driven by individual trait changes, which would be missed by the univariate analysis.

## 3. Conclusion and future directions

By comprehensively examining the relationships between gestation length, body mass and lifespan across 845 eutherian mammals (approx. 14% of extant species [59]), we reconstructed the tempo and mode of gestation length evolution. While lifespan and gestation length are correlated with each other and with body mass, studying them together allows us to explore broader patterns of life-history evolution in mammals. We found evidence for numerous evolutionary shifts in the gestation length optima at the origins of diverse taxa; some of these shifts were linked to changes in body mass and lifespan phenotypic optima and associated with major ecological transitions (e.g. the terrestrial to marine transition at the base of the Cetacea and Pinnipedia clades and the evolution of flight at the base of the Chiroptera). By considering multiple traits simultaneously, we aim to uncover potential interrelationships and trade-offs that shape reproductive strategies.

While our analyses yield valuable insights into the evolutionary history of gestation length adjusted for body mass and lifespan, building upon previous work based on fewer taxa and different methodologies [1,4,7,8,12,14,15,25,40], we also acknowledge potential caveats and limitations. Aiming to maximize the number of mammalian species included in our study, our analyses of gestation length considered data for the body and only one life-history trait, lifespan. There are potentially several additional life-history traits that may have covaried with gestation length, influencing its evolution, such as litter size, neonate developmental status and mating system [1,4,26,28]. Unfortunately, data for many life-history traits remain scarce, so their inclusion would have dramatically reduced the number of taxa in our analyses. Additionally, we note that research efforts may influence the values of the traits in taxa that are better studied, potentially biasing our understanding of their relationships [60,61]. Furthermore, we note that gestation length is not easily detectable from the fossil record, making it difficult to validate predicted evolutionary shifts by its examination.

The evolution of gestation length is associated with the evolution of body size and/or lifespan in some taxa and not associated in others (see also [4]), a finding that has implications for the genetic loci that contribute to the observed variation of gestation length in different mammalian clades and their effect sizes. For example, variation in gestation length is strongly associated with variation in body size in Primates (electronic supplementary material, table S2). This finding is consistent with human genome-wide association studies of gestation length, where some of the genetic variants that influence gestation length are also known to influence birth weight [29,62]. However, the lack of association between gestation length and body size in many other eutherian taxa (see also [4]) raises the hypothesis that the genetic loci (and/or their effect sizes) for gestation length in these taxa will also differ. Consequently, we might expect correlational selection (i.e. selection favouring correlations between interacting traits) in taxa where gestation length coevolves with body mass and lifespan but lack of correlational selection in lineages where the traits evolve independently of each other.

## 4. Methods

### (a) Data collection

We retrieved data on gestation length, lifespan and body mass for 845 representative extant species of eutherian mammals. This information was sourced from four databases: PanTHERIA [33], AnAge [34], EltonTraits [35] and MOM-Mammals [36]. Given that life-history traits are highly correlated with each other, our rationale was to include one additional and well-studied life-history trait so that we can examine the correlation between gestation length and other life-history traits (but without reducing the taxon sample size of our dataset, which would happen if we included several additional life-history traits). To ensure the quality of our data, we only included values for adult individuals of each species and conducted a meticulous manual inspection to resolve any discrepancies across databases. If discrepancies were found, our procedure involved cross-referencing the data with original sources. Before analysis, we naturally log-transformed all continuous predictor variables to reduce skewness and improve the accuracy of our findings. The study employed a consensus phylogeny obtained from the supertree reconstructed by Upham *et al*. [37]. Briefly, the researchers first constructed a supermatrix of 31 genes from 4098 species and inferred the maximum likelihood phylogeny and dates of divergence (using fossil calibrations). They then added to this backbone phylogeny several species-level ‘patch’ phylogenies, each of which contained species from a single representative lineage in the backbone phylogeny. This brought the total number of eutherian species in the phylogeny to 5099. This robust framework allowed us to account for evolutionary relationships among the species in our analysis. Our dataset is shown in electronic supplementary material, data S1.

### (b) Phylogenetic regression

Comparisons among species, owing to their shared ancestry, violate the assumption that data points are independently drawn by a common distribution [16]. To account for this lack of independence, we employed PGLS regression analyses using the ‘gls’ function in the R package nlme [63]. We used PGLS to examine the relationships of gestation length with body mass and lifespan (log(gestation length) ~ log(body mass) + log(lifespan) + log(body mass × lifespan)) and of gestation length with body mass (log(gestation length) ~ log(body mass)) in eutherian mammals. First, we categorized the 845 eutherian mammals in our dataset into 16 clades based on their taxonomy. The 16 taxa were Afrosoricidae, Artiodactyla (excluded Cetacea), Carnivora, Cetacea, Cingulata, Eulipotyphla, Lagomorpha, Chiroptera (Yangochiroptera and Yinpterochiroptera), Perissodactyla, Pilosa, Pinnipedia, Rodentia and Primates (NWM, OWM and Prosimians). We analysed NWM and OWM separately because they represent two major monophyletic groups within primates. These groups differ genomically, in their geographic distributions, evolutionary history, anatomy and behaviour. In contrast, the group defined by OWM + NWM is paraphyletic, since it excludes the hominids [64,65]. For similar reasons, we analysed Yangochiroptera and Yinpterochiroptera separately [66–68].

Next, we fitted multiple models, and we calculated the Bayesian information criterion (BIC) for Pagel’s *λ* [23] and Ornstein–Uhlenbeck (OU) models [17]. To ensure robustness, we performed 500 iterations, initializing the starting values for each model in the range of 0 to 1. Pagel’s *λ* [23] model was applied using the ‘corPagel()’ function, and the OU model was applied using the ‘corMartins()’ function, both from the nlme [63] package in R. Subsequently, we selected the best-fitting model for each mammalian group based on the BIC, choosing models where the absolute BIC difference was 5 or greater [69] (electronic supplementary material, tables S2 and S4). To evaluate multicollinearity, we examined the VIF in our models, using the function VIF() from the regclass [70] package in R. In general, VIF values of 1 indicate the absence of multicollinearity, while values exceeding 5 raise concerns about potential multicollinearity bias in ecological datasets [38,39]. To facilitate the interpretation of the interactions in our model and reduce multicollinearity, we centred the predictor variables, body mass and lifespan, by subtracting their mean values [71]. This adjustment ensures that the main effects are interpretable as the effect of each predictor when the other is at its average value. Centring is crucial for meaningful interpretation of the interaction term (body mass × lifespan) in our phylogenetic generalized least squares (PGLS) regression analyses.

### (c) Ancestral reconstruction of relative gestation length

We performed ancestral reconstruction of gestation length after statistically controlling for the effects of body mass and lifespan. We first fitted a model with gestation length as the dependent variable and body mass, lifespan and their interaction term (body mass × lifespan) as independent variables, allowing us to isolate the influence of gestation length independent of body size and lifespan. Specifically, we extracted the residuals of gestation length while controlling for body mass and lifespan (employing the model ‘log(gestation length) ~ log(body mass) + log(lifespan) + log(body mass × lifespan)’), using the function fastAnc in the package phytools v.0.7 [72]. It is important to note that the use of residuals may affect the subsequent analyses [73]. We next used ancestral state reconstruction to estimate the evolutionary history of this relative gestation length by mapping the residual values from the phylogenetic regression onto the mammalian phylogeny using the R packages ggtree v. 2.4.0 [74] and phytools v. 0.7 [72].

### (d) Identifying evolutionary shifts in gestation length

To detect evolutionary shifts in gestation length across eutherian mammals, we used the R package bayou v.2.2.0 [43]. This tool employs a Bayesian reverse-jump Markov Chain Monte Carlo (MCMC) approach, allowing multiple optima under the OU model, identifying the number, the magnitude and the location of the shifts. We implemented this approach by combining three parallel chains of five million iterations with a burn-in proportion of 0.3. We allowed only one shift per branch, and the total number of shifts was calculated based on the conditional Poisson prior with a mean of 2.5% of the total number of branches in the tree and a maximum number of shifts equal to 5%, following the authors’ recommendations [75]. For all the other parameters, we used the recommended distributions in the publicly available tutorial at https://github.com/uyedaj/bayou/blob/master/tutorial.md. The MCMC was initialized with randomly selected parameters for the first 1000 generations to improve the fit of the model. Finally, we ensured that independent chains had converged on similar regions in the parameter space by Gelman’s [76] R for log-likelihood and *σ*^2^ (electronic supplementary material, figure S2).

### (e) Evolutionary shifts of gestation length, body mass and lifespan across eutherian mammals

To investigate evolutionary shifts in the evolution of all three life-history traits across the eutherian mammal phylogeny, we used the PhylogeneticEM [42,58] R package. This method infers evolutionary shifts in multivariate correlated traits on phylogenies via an OU process. Shift positions were estimated using the expectation–maximization (EM) algorithm, considering varying numbers of unknown shifts, and the optimal number of shifts was determined using a lasso-regression model selection procedure. All parameters were kept at their default settings except for the maximum number of shifts, which was set to 18.

## Data Availability

All data, scripts and supplementary information associated with this manuscript are publicly available on the Dryad Digital Repository [77]. Supplementary material is available online [78].
